# Saint Valentine and Epilepsy: from Medieval Devotion to Modern Global Advocacy

**DOI:** 10.1055/s-0046-1818611

**Published:** 2026-04-07

**Authors:** Patricia Vilarinho Tambourgi, Li Min Li, Josemir W. Sander

**Affiliations:** 1 Universidade Estadual de Campinas, Faculdade de Ciências Médicas, Departamento de Neurologia, Campinas SP, Brazil.; 2 UCL Queen Square Institute of Neurology, London, United Kingdom.

**Keywords:** Epilepsy, Health Education, History, Medieval, Religion and Medicine, Saints

## Abstract

Epilepsy, described for millennia, has historically been interpreted through supernatural perspectives, leading to the search for religious intercession. Among the saints associated with the disease, Saint Valentine stood out in medieval Europe, especially in German-speaking regions, where his image was depicted curing people with seizures. Pilgrimages to sites with relics and popular names such as “Saint Valentine's Disease” highlight this devotion. In the 21 ^st^ century, this symbolic heritage was reinterpreted in the context of advocacy, influencing the creation of International Epilepsy Day, celebrated on the second Monday of February, close to the saint's liturgical date on February 14 ^th^ . This article revisits the historical trajectory of this association and discusses how cultural elements can be used to reduce stigma and promote scientific awareness about epilepsy.

## INTRODUCTION

Epilepsy is one of the oldest medical conditions described in human history, with reports dating back millennia. 1 2 Before the consolidation of neurological knowledge, seizures were often interpreted as divine manifestations or signs of possession, prompting ritualized responses and the search for religious intercession. 3 4 In this context, various saintly figures were recognized as protectors against epileptic seizures. Among them, Saint Valentine is one of the most notable. 5 6

Saint Valentine of Terni, also known as Valentine of Interamna, was a third-century bishop and martyr traditionally associated with the Umbrian city of Terni, in central Italy. According to hagiographical sources, 5 6 he was consecrated as bishop at a young age and became known for his pastoral care, missionary activity, and reputed healing abilities during a period marked by political instability and recurring persecutions of Christians by the Roman Empire. His martyrdom, conventionally dated to 269 AD, occurred in Rome, where he was executed for practicing his faith. Following his death, his cult grew rapidly in the regions of Umbria and Lazio and, later, spread across continental Europe as relics were translated and local devotional traditions developed.

From the late Middle Ages onwards, Saint Valentine's iconography increasingly portrayed him as a healer. He was frequently shown placing his hands upon individuals experiencing involuntary movements or loss of consciousness, features retrospectively compatible with epileptic seizures. 5 Medieval miracle collections and regional devotional records from central and northern Italy, southern Germany, and parts of the Austrian Alps describe petitions to Saint Valentine for protection against “falls”, “fits”, or “the falling sickness.” In several German-speaking areas, epilepsy became popularly known as “Saint Valentine's disease,” reflecting the depth of this association within folk practice and liturgical commemoration. Pilgrimages to churches holding his relics, particularly in Terni and in the Rhineland, were common between the 15 ^th^ and 18 ^th^ centuries, and devotional rituals often included blessings, contact with reliquaries, or participation in feast-day processions.

**Figure 1:**
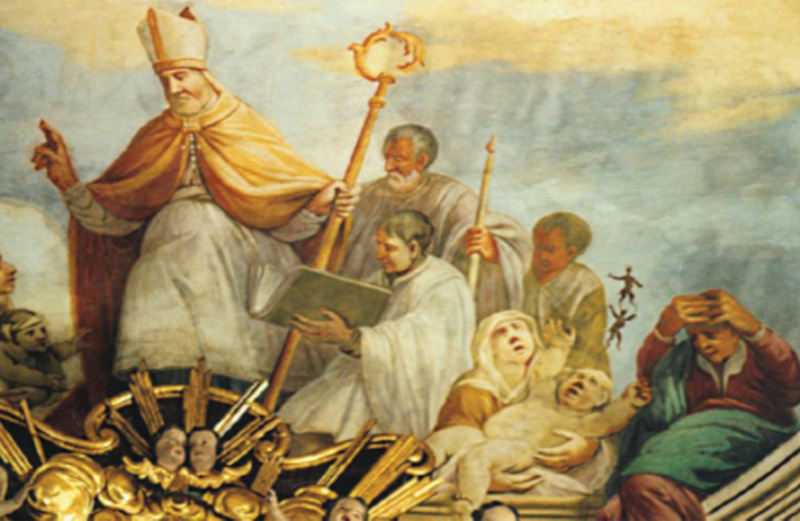
Saint Valentine, ceiling fresco, Unteileterbach, Germany (1740). 5 The fresco depicts Saint Valentine assisting individuals suffering from various ailments. At the center, a mother holds an infant with raised arms, splayed legs and an open mouth, the abnormal posturing suggestive of West syndrome. Small fleeing figures interpreted as spirits symbolize the expulsion of the “falling sickness,” reflecting early modern beliefs about the saint's intercessory power in epilepsy.

The link between Saint Valentine and epilepsy appears to have been reinforced by the broader medieval understanding of seizures as events with spiritual or supernatural dimensions. In this context, saints renowned for compassion, healing or intercession in bodily afflictions were frequently invoked. Saint Valentine's reputation for restoring physical and emotional well-being, combined with local testimonies of healing at his shrines, strengthened his role as a protector of those affected by convulsive disorders. As suggested, 5 visual depictions of the saint treating individuals with seizure-like symptoms ( Figure 1 ) persisted for centuries, illustrating the durability of this devotional pattern across Europe.

The rise of modern neurology in the 19 ^th^ century reframed epilepsy within a scientific and medical paradigm, but its symbolic association with Saint Valentine has endured. This historical legacy has contributed to contemporary reflections on how cultural narratives and devotional imagery have shaped societal responses to the condition. The persistence of Saint Valentine's name in connection with epilepsy, in historical texts and in contemporary advocacy contexts, illustrates the long-standing human search for meaning, protection, and compassion in relation to seizure disorders.

Other traditions also associated saints with epilepsy. In Italy, Saint Bibiana; in Scandinavia, Saint Bridget; and in Spain, Saint Vincent Ferrer. Figures such as Saint Vitus, Saint Dymphna, and even the Magi were, at specific points in medieval history, also considered protectors of people with epilepsy. 3

These devotions varied according to culture and reveal the ubiquity of spiritual recourse in the face of medical ignorance before the modern era. 4 With the advancement of medical knowledge and the professionalization of care, the religious role was gradually replaced by evidence-based clinical practices. However, symbolic elements persisted and were incorporated into awareness initiatives.

There are three international dates that stand out in the contemporary advocacy calendar in Brazil.

### Latin American epilepsy day

This date was established on the 9 ^th^ of September 2000, at the 1 ^st^ Latin American Epilepsy Congress, held in Santiago, Chile, as a regional landmark for education and mobilization. 7 The Demonstration Project on Epilepsy carried out by Assistência à Saúde de Pessoa com Epilepsia (ASPE) 8 established this as Brazil's national date for epilepsy awareness campaigns. This was then adopted by the Associação Brasileira de Educação (ABE) and Federação Brasileira de Epilepsia (EpiBrasil), two national lay associations, as well as the Brazilian League Against Epilepsy. 9

### Purple day

Created in 2008 by Cassidy Megan in Canada, setting the 26 ^th^ of March as a global awareness date. The color purple, associated with the lavender flower, symbolizes empathy and the fight against social isolation. In Brazil, with a national date for epilepsy awareness already established on September 9 ^th^ , Purple Day added an important new dimension to epilepsy advocacy. Taking place in the first semester, it strategically complemented Brazil's National Epilepsy Day in the second semester, ensuring broader visibility and sustained engagement throughout the year. The color purple was rapidly embraced by existing social movements, particularly among youth and women.

### International epilepsy day

Created by the International League Against Epilepsy (ILAE) and the International Bureau for Epilepsy (IBE), it is celebrated on the second Monday of February. 10 11 European proposals influenced the choice in the 2010s, when seeking a date for a European Epilepsy Day. Discussions on this continental date began with the perception of the need for a figure that could symbolize and provide a reference for the date. At the time, the Irish group was particularly active and was responsible for bringing Saint Valentine to the fore, an idea that was well received. Initially, the proposal was for a week of activities around the saint's date.

During the European Congress, to avoid direct conflict with Valentine's Day, a motion was made a date around the 14 ^th^ , defining Epilepsy Day as the second Monday in February. The proposal was officially presented in a 2011 White Paper. 12 From a European initiative, the idea went global, and it was agreed the date would become an international day epilepsy. This was in light of the perception that a worldwide awareness date had yet to be established, which could be celebrated simultaneously across the planet.

In Brazil, International Epilepsy Day has been adopted, albeit with lesser intensity, compared to the other two dates, as it was recently established. It coincides with the summer holidays and is close to Carnaval, a national festivity.

In contemporary practice, these commemorative dates remain highly relevant, as they provide recurring opportunities to enhance public understanding, reduce stigma, and promote equitable access to epilepsy care. 13 Awareness campaigns held on regional and global days, such as Latin American Epilepsy Day, Purple Day, and International Epilepsy Day, act as catalysts for disseminating evidence-based information and encouraging early diagnosis and treatment adherence. They also mobilize support organizations, healthcare professionals, policymakers, and the general public around coordinated initiatives that highlight both the medical and social dimensions of the disease.

By sustaining visibility throughout the year, these dates help counter persistent misconceptions about epilepsy and contribute to a cultural environment in which individuals with epilepsy can seek care and social participation without discrimination. Their continued use demonstrates how collective advocacy can translate historical narratives into meaningful public health impacts. Reviews of awareness campaigns document their positive effect on the visibility of epilepsy and public education, although they emphasize the need for impact assessment and cultural adaptation. 14

## FINAL CONSIDERATIONS

The trajectory of Saint Valentine as the patron saint of epilepsy illustrates the persistence of cultural narratives that have shaped the social understanding of seizures. His symbolic reappearance in the context of modern global advocacy shows how historical elements can be reused for public mobilization, contributing to stigma reduction and the promotion of medical knowledge. The articulation between cultural memory and public health strategies offers a powerful avenue for building a more inclusive and informed discourse on epilepsy.
